# Transcriptomic analysis reveals novel hub genes associated with astrocyte autophagy in intracerebral hemorrhage

**DOI:** 10.3389/fnagi.2024.1433094

**Published:** 2024-07-03

**Authors:** Yun Zheng, Chengwei Duan, Haoyun Yu, Guannan Jiang, Haitao Shen, Haiying Li, Zongqi Wang, Xiaohan Zhou, Xiang Li, Mingqing He

**Affiliations:** ^1^Department of Geriatrics, The First Affiliated Hospital of Soochow University, Suzhou, China; ^2^Medical Research Center, Affiliated Hospital 2 of Nantong University, Nantong, China; ^3^Soochow Medical College of Soochow University, Suzhou, China; ^4^Department of Neurosurgery & Brain and Nerve Research Laboratory, The First Affiliated Hospital of Soochow University, Suzhou, China; ^5^Institute of Stroke Research, Soochow University, Suzhou, China

**Keywords:** biomarkers, autophagy, intracerebral hemorrhage, astrocyte, lipopolysaccharide

## Abstract

**Introduction:**

Neuroinflammation serves as a critical local defense mechanism against secondary brain injury following intracerebral hemorrhage (ICH), and astrocytes play a prominent role in this process. In this study, we investigated astrocytic changes during the inflammatory state after ICH to identify new targets for improving the inflammatory response.

**Methods:**

We stimulated mouse astrocytes with lipopolysaccharide (LPS) *in vitro* and analyzed their transcriptomes via ribonucleic acid sequencing. We created an ICH model in living organisms by injecting autologous blood.

**Results:**

RNA sequencing revealed that 2,717 genes were differentially expressed in the LPS group compared to those in the saline group, with notable enrichment of the autophagic pathway. By intersecting the 2,717 differentially expressed genes (DEGs) with autophagy-related genes, we identified 36 autophagy-related DEGs and seven hub genes. Previous studies and quantitative reverse transcription-polymerase chain reaction results confirmed the increased expression of phosphatidylinositol 3-kinase catalytic subunit type 3 (Pik3c3), AKT serine/threonine kinase 1 (Akt1), and unc-51 like autophagy activating kinase 2 (Ulk2) in astrocytes after ICH. Transcription factors and target miRNAs were identified for the final three DEGs, and 3-methyladenine and leupeptin were identified as potential therapeutic agents for ICH.

**Conclusion:**

Our findings suggest that astrocyte autophagy plays a critical role in ICH complexity, and that Pik3c3, Akt1, and Ulk2 may be potential therapeutic targets.

## 1 Introduction

Intracerebral hemorrhage (ICH) is a serious condition that frequently results in death and leads to significant disability. It constitutes approximately 15–20% of the total stroke burden and exhibits a fatality rate of approximately 50% among stroke-related mortalities. Annually, ICH causes approximately 2.8 million deaths globally (Schrag and Kirshner, 2020). There are two main types of brain damage associated with ICH—primary and secondary. Primary brain injury occurs because of mechanical damage caused by the formation and subsequent expansion of a hematoma following ICH. This expansion leads to compression of the surrounding tissues (Cao et al., 2016). Secondary brain injury (SBI) involves several pathological mechanisms. It arises from the release of metabolic byproducts from the hematoma, triggering cellular toxic reactions, inflammation, oxidative stress, and other signaling cascades. Consequently, cerebral edema, disruption of the blood-brain barrier, and neuronal apoptosis result, collectively referred to as SBI, occur (Xi et al., 2006; Ziai, 2013; Zhu et al., 2019; Xue and Yong, 2020). However, surgical removal of hematomas, is not widely performed in most patients with ICH. This is because the clinical effectiveness of the procedure is inconclusive and potentially has negative consequences (Hemphill et al., 2015). Consequently, studies have increasingly focused on SBI as a significant contributing factor to neurological deficits following ICH (Chen et al., 2015).

Previous studies have underscored the significance of neuroinflammation as a crucial mechanism underlying secondary damage following ICH (Xi et al., 2006; Shi S. X. et al., 2020). Microglia and astrocytes are postulated to act as the major inflammatory cells during blood exudation (Wang, 2010). Studies have shown that in the early stages of cerebral hemorrhage, significant astrocyte activation occurs in the space around the hematoma (Han et al., 2016). The number of activated astrocytes initially increases on the first day and remains elevated until the seventh day (Lan et al., 2017). Astrocytes are predominant cellular components of the central nervous system (CNS). These processes include neurotransmitter uptake and metabolite recycling to support active neurons (Nedergaard et al., 2003; Alvarez et al., 2013). They exhibit high sensitivity to changes in the surrounding environment and display reactive hyperplasia, which is characterized by cellular hypertrophy and rapid proliferation, and are referred to as reactive astrocytes (Choudhury and Ding, 2016; Pekny et al., 2016). After ICH, astrocytes secrete a diverse array of cytokines and chemokines that potentially facilitate microglial polarization and play a vital role in the development of ICH pathogenesis (Lan et al., 2017).

This study used lipopolysaccharide (LPS)-stimulated astrocytes as a model to investigate the changes occurring in astrocytes under inflammatory conditions. Bioinformatic analysis revealed that autophagy was significantly activated in astrocytes during neuroinflammation. Autophagy is an intracellular degradation and recycling mechanism that allows cells to break down and remove damaged or surplus organelles, proteins, and other macromolecules, thereby maintaining cellular homeostasis and balance (Glick et al., 2010; Liu et al., 2023). Research has shown that autophagy helps maintain homeostasis in ICH by clearing damaged proteins and organelles. For example, heme and iron can exacerbate SBI by enhancing autophagy (Fu et al., 2022). Accumulating evidence has indicated that autophagy is activated and plays a role in the pathophysiology of ICH-induced SBI. Thus, the mechanisms of autophagy and inflammation in ICH are closely intertwined, and the regulation of autophagy in ICH-induced SBI inflammation is an intriguing hypothesis. By examining the expression of autophagy-related factors in astrocytes under inflammatory conditions following ICH, we aimed to identify key factors that could lead to the development of new therapeutic strategies for treating ICH. This approach may provide novel insights into improving outcomes after ICH.

## 2 Materials and methods

### 2.1 Ethics and animals

The Chinese Academy of Sciences (Shanghai, China) provided adult male C57BL6/J mice weighing 20–25 g. During the experiment, they were kept in a temperature-controlled room at 23°C with 40% humidity and subjected to a light-dark cycle simulating natural day-night rhythms (lights were turned on at 7 p.m.). Food and water were provided to the animals *ad libitum*, and the study protocol was approved by the Ethics Committee of the First Affiliated Hospital of Soochow University.

### 2.2 *In vivo* ICH model

As described in previous studies mice were initially anesthetized with 3% isoflurane and a 67%:33% N_2_O/O_2_ gas mixtures until they became unresponsive to tail pinching (Yang et al., 2020; Zhu et al., 2020). To maintain anesthesia, the mice were placed on a stereotaxic frame (RWD; Shenzhen, China) and administered 1.5% (vol/vol) isoflurane through a nose cone. Using dental instruments, a burr hole was meticulously drilled at precise anterior and lateral coordinates of the bregma. After this, the right caudate nucleus was infused with 30 mL blood, at a rate of 3 μL/min, using 26-gauge needles. Following completion of the infusion, the needle was left in place for a 10 min before slowly retracting at a rate of 1 mm/min. Subsequently, the mice were allowed to gradually recover in a temperature-controlled enclosure. In sham-operated mice, no injections were administered during the surgical procedures.

### 2.3 Astrocyte isolation

Using magnetic-activated cell sorting, astrocytes were isolated from the brain tissues of mice following macroscopic dissection (Holt and Olsen, 2016). Tissue from the perihematomal region was harvested 24 h post-modeling in the ICH group, while the same corresponding area was collected in the sham group. The samples were then enzymatically dissociated using the Neural Tissue Dissociation Kit (P) from Miltenyi Biotec (Cologne, Germany). Myelin and cell debris were removed using the Myelin Removal Beads Kit (Miltenyi Biotec). Astrocytes were isolated using Anti-ACSA-2 microbeads (Miltenyi Biotec). All separation steps were performed using an autoMACS Pro Separator (Miltenyi Biotec).

### 2.4 *In vitro* cell culture

The mouse astrocyte cell line was obtained from the American Type Culture Collection (ATCC, Manassas, VA, United States). Mouse astrocytes were separated 24 h after ICH molding in complete DMEM medium supplemented with 100 U/mL penicillin, 100 g/mL streptomycin, and 10% fetal bovine serum (Gibco, Carlsbad, CA, United States). Optimal conditions were maintained by renewing the culture medium every 3 days. To prevent phenotypic changes due to extended passage durations, the cell line was only subjected to only 20 passages.

### 2.5 *In vitro* inflammation model

Lipopolysaccharide (LPS) from *Escherichia coli* (Sigma-Aldrich, Cat No. L2630) was dissolved in phosphate-buffered saline (PBS) for *in vitro* experiments. Astrocytes were stimulated with either saline or LPS (100 ng/mL). After 24 h, the astrocytes were harvested.

### 2.6 RNA sequencing

Sequencing was conducted using Zhongke Gene Biotechnology (Jiangsu, China). Astrocytes were obtained from two groups—saline and LPS (100 ng/mL). TRIzol reagent was used for RNA extraction, and the concentration and purity of the extracted RNA were determined using Nanodrop2000. The integrity of total RNA was evaluated using cryogenic agarose gel electrophoresis and a bioanalyzer (Agilent 2100). A strand-specific library was constructed using the Illumina TruSeq RNA Sample Prep Kit, specifically the TruSeq Stranded Total RNA Human/Mouse/Rat Kit (RS-122-2202) and the TruSeq Small RNA Library Prep Kit (RS-200-0012). Library construction required a minimum total quantity of 5 μg RNA, a concentration of at least 200 ng/μL, and an optical density (OD) 260/280 ratio between 1.8 and 2.2. Subsequently, complementary DNA (cDNA) library construction involved ribosomal RNA (rRNA) removal, messenger RNA (mRNA) fragmentation, reverse transcription to synthesize cDNA in a strand-specific manner, and adapter ligation, followed by the digestion of double-stranded cDNA using the uracyl N-glycosylase (UNG) enzyme. After establishing the cDNA library, polymerase chain reaction (PCR) amplification was performed prior to conducting 2 × 150 bp sequencing on an Illumina sequencing platform (Illumina, California, United States).

### 2.7 RNA-seq analysis

The *M. musculus* reference genome (GRCm39.105) was downloaded from the Ensembl website.^1^ For differential expression analysis, raw counts were normalized using the DEseq2 package (Love et al., 2014). Using MetaAnalyst 5.0 (Pang et al., 2021), the data were analyzed via principal component analysis (PCA). All statistical analyses were performed using R, version 4.2.2. Multiple hypothesis correction was performed using the Benjamini–Hochberg method, with *p*_adj_ = 0.05, and | log2 (foldchange) | >1 serving as the thresholds. A volcano plot was generated using ggplot2 package (Wickham, 2016). Heatmaps were generated using the pheatmap package, wherein the *Z*-scores were computed after hierarchical clustering, affecting only visualization. In each pairwise comparison, clusterProfiler (Yu et al., 2012) was employed to identify enriched Gene Ontology (GO) categories among the significant genes, whereas the database for annotation, visualization, and integrated discovery database was utilized for Kyoto Encyclopedia of Gene and Genome (KEGG) pathway enrichment analysis. Bar and bubble plots were created using the ggplot2 package. Additionally, we conducted gene set enrichment analysis (GSEA), which ranks genes based on their differential expression between two sample groups and then determined whether the predefined gene sets were enriched at the top or bottom of the ranking list (Subramanian et al., 2005). In this study, we used a predefined gene set, c2.cp.kegg.v7.0.symbols.gmt. The datasets generated and/or analyzed during the current study have been deposited in the National Center for Biotechnology Information (NCBI) and are publicly available under the following accession number: PRJNA1067976.

### 2.8 Identification and analysis of differentially expressed autophagy-related genes

Using the DEseq2 package (Love et al., 2014) differentially expressed genes (DEGs) among the samples treated with LPS and saline were identified in the dataset. A set of 142 autophagy-associated genes was obtained from the KEGG database.^2^ A Venn diagram was generated using the Evenn platform^3^ to obtain 36 autophagy-related DEGs. Gene expression levels were visualized using heatmaps generated with the pheatmap R package. Enrichment analyses for the GO and KEGG pathways were performed following standard procedures as previously outlined.

### 2.9 Bioinformatics analysis of differentially expressed autophagy-related genes

According to the Search Tool for the Retrieval of Interacting Genes/Proteins (STRING) database (Szklarczyk et al., 2015), protein–protein interaction (PPI) networks were formed by considering interactions with a score greater than 0.4 as being statistically significant. To detect the central genes, we employed the CytoNCA plugin (Tang et al., 2015) of the Cytoscape software V3.9.1 (Shannon et al., 2003). Hub genes were subjected to functional enrichment analysis using GeneMANIA (Warde-Farley et al., 2010). However, PCR verification was performed for all seven hub genes, and it was found that only Akt1, Pik3c3, and Ulk2 were significantly upregulated in astrocytes after ICH. Therefore, we further analyzed these three factors. We used the Transcriptional Regulatory Relationships Unraveled by Sentence-based Text mining database to predict transcription factors, microRNA-target interactions database (miRTarBase) to identify upstream miRNAs, and drug signatures database (DSigDB) to discover small-molecule drugs associated with these three differentially expressed autophagy-related genes. These analyses were performed using the Enrichr platform as described by Yerramsetty et al. (2011).

### 2.10 Correlation analysis

We employed a linear correlation analysis to assess the association between the three upregulated autophagy-related factors, Pik3c3, Akt1, and Ulk2, and the phenotypes of A1 and A2 type astrocytes. Spearman’s correlation tests were used for this analysis. Furthermore, to visualize the correlation heatmap, we utilized the “corrplot” package, and for generating graphical representations of linear correlation analysis, we utilized the cor.test function within the stats package.

### 2.11 Immunofluorescence staining

Immunofluorescence staining was performed on frozen sections to investigate protein expression in astrocytes and the phenotypic changes surrounding the hematoma. Briefly, brain tissues were fixed in a 4% paraformaldehyde solution, embedded in optimal cutting temperature (OCT) compound and sliced into sections with a thickness of 10 μm. After three washes with PBS, the sections were blocked with immunostaining blocking buffer (Beyotime) at room temperature (RT) for 1 h. Subsequently, overnight incubation at 4°C was conducted with primary antibodies: mouse anti-GFAP (1:300 dilution, Cat No. ab 279289, Abcam), rabbit anti-C3 (1:200 dilution, Cat No. ab 181147, Abcam). After three more washes with PBS, the sections were then incubated at RT for 1 h with donkey anti-mouse IgG Alexa Fluor 555 (1:300 dilution, A-32773, Invitrogen) and donkey anti-rabbit IgG Alexa Fluor 488 (1:300 dilution, A-21206, Invitrogen). Subsequently, 4′,6-diamidino-2-phenylindole (DAPI) counterstaining was performed (Sangon Biotech, Shanghai, China, cat no. E607303), and the sections were analyzed using a U-RFL-T fluorescence microscope (Nikon, Tokyo, Japan). At least six sections were randomly selected from each sample for examination. The number of cells showing co-localization of a green GFAP-labeled fluorescent cell body with red fluorescence was quantified as positive for both GFAP and C3. Cell positivity was determined by cell counting using the ImageJ software (NIH, United States). All analyses were conducted in accordance with the principles of blinding.

For cell staining, consistent with brain tissues staining, except for fixation with ice-cold ethanol for 15 min, the following antibodies were used: mouse anti-GFAP (1:300 dilution, Cat no. ab 279289, Abcam), and donkey anti-mouse IgG Alexa Fluor 555 (1:300 dilution, A-32773, Invitrogen). Under the microscope, we observed that the astrocytes and nuclei completely overlapped, indicating that the astrocytes were successfully separated and there was no contamination.

### 2.12 Quantitative reverse transcription-polymerase chain reaction

Total RNA was extracted using the TRIzol reagent (Thermo Fisher Scientific Shanghai, China), followed by reverse transcription into cDNA using the PrimeScript RT reagent kit (Thermo Fisher Scientific, Shanghai, China) according to the manufacturer’s instructions. PowerUp^™^ SYBR^™^ Green (Applied Biosystems^™^) was utilized for quantitative reverse transcription-polymerase chain reaction (qRT-PCR) analysis on the LightCycler 480 System (Roche). β-actin (GenScript, China) served as the positive control, and the Livak method (2^−ΔΔCt^ method) was utilized to determine the relative changes in gene expression. The following PCR primer sequences were used for target molecules: Ulk2: 5′-AGCTTCAGCATGAAAAC ATCGT-3′ (forward) and 5′-CGATTGGCATAAGACAACAGGA-3′ (reverse); Akt1: 5′-ATGAACGACGTAGCCATTGTG-3′ (forward) and 5′-TTGTAGCCAATAAAGGTGCCAT-3′ (reverse); Pik3c3: 5′-CCTGGACATCAACGTGCAG-3′ (forward) and 5′-TGTCT CTTGGTATAGCCCAGAAA-3′ (reverse); Pten: 5′-TTTGCTA GTGAGTGGAATCCTCT-3′ (forward) and 5′-TGTGACAAAAGT GACACAGATCA-3′ (reverse); Atg5: 5′-TGTGCTTCGAGATGTG TGGTT-3′ (forward); and 5′-GTCAAATAGCTGACTCTTGGCAA-3′ (reverse); Atg7: 5′-GTTCGCCCCCTTTAATAGTGC-3′ (forward) and 5′-TGAACTCCAACGTCAAGCGG-3′ (reverse); Rab7: 5′-AGGCTTGGTGCTACAGGAAAA-3′ (forward) and 5′-CTTG GCCCGGTCATTCTTGT-3′ (reverse); β-actin: 5′-TCAGCAAG CAGGAGTACGATG-3′ (forward) and 5′-GTGTAAAACGCAG CTCAGTAACA-3′ (reverse).

### 2.13 Statistical analysis

Unless otherwise specified, data are presented as mean ± standard deviation (SD). Group comparisons were performed using student’s *t*-test. Statistical analyses were performed using the GraphPad Prism software (version 9.4.1; GraphPad, United States). Normality distribution of the dataset was assessed using the Kolmogorov–Smirnov test, with statistical significance defined as a *p*-value <0.05.

## 3 Result

### 3.1 RNA-seq and data preprocessing

**Figure 1:**
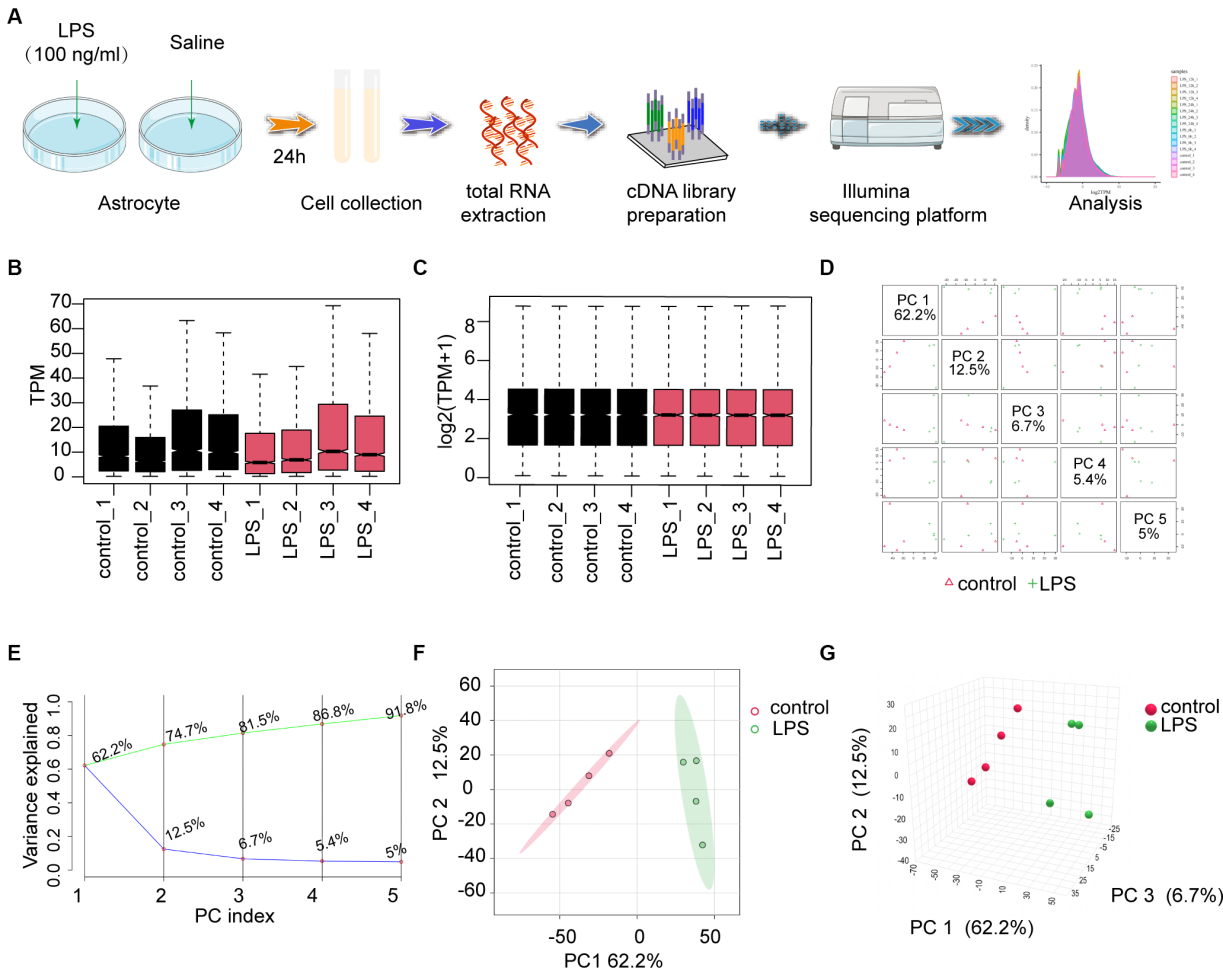
The workflow and preprocessing of RNA-seq data sets. **(A)** Analytic workflow of RNA-seq. Relevant details can be accessed in the materials and methods section. **(B,C)** Comparing gene expression levels between different samples before and after normalization is shown by a box plot. **(D)** A summary of the top 5 primary components in PCA. **(E)** Scree plot of the PCA. **(F,G)** Revealing distinct distribution patterns, the samples exhibited diverse arrangements through both 2D- and 3D-PCA-plots. PCA, principal components analysis.

RNA sequencing (RNA-seq) was performed on astrocytes treated with LPS (100 ng/mL) or saline (Figure 1A). Box plots were used to demonstrate the varying dispersion of gene expression levels among different specimens (Figure 1B). To address this, a normalization function was applied to data processing (Figure 1C). Subsequently, PCA was conducted to investigate the differences between various groups. PCA is a dimensionality reduction technique used to simplify and visualize high-dimensional data. By applying PCA, we can intuitively observe the relationships between samples. PCA generates multiple principal components (PCs), each representing a linear combination of the original data. For example, the first principal component (PC1) explains the maximum variance, and the second principal component (PC2) explains the next largest variance. In a PCA plot, each point represents a sample, and its coordinates indicate the projection of the sample in the principal component space. The top five PCs were used to create pairwise score plots, which collectively accounted for 91.8% of the variance in all variables (Figure 1D). The explained accumulated variance explained is depicted by the green line, whereas the blue line represents the variance explained by the individual PCs (Figure 1E). In this study, both two-dimensional and three-dimensional PCA plots were used to verify and visualize clustering within the sample groups (Figures 1F,G). These figures demonstrate that our sample groupings exhibited good clustering.

### 3.2 Autophagy-related pathways are significantly activated after LPS stimulation in astrocytes

**Figure 2:**
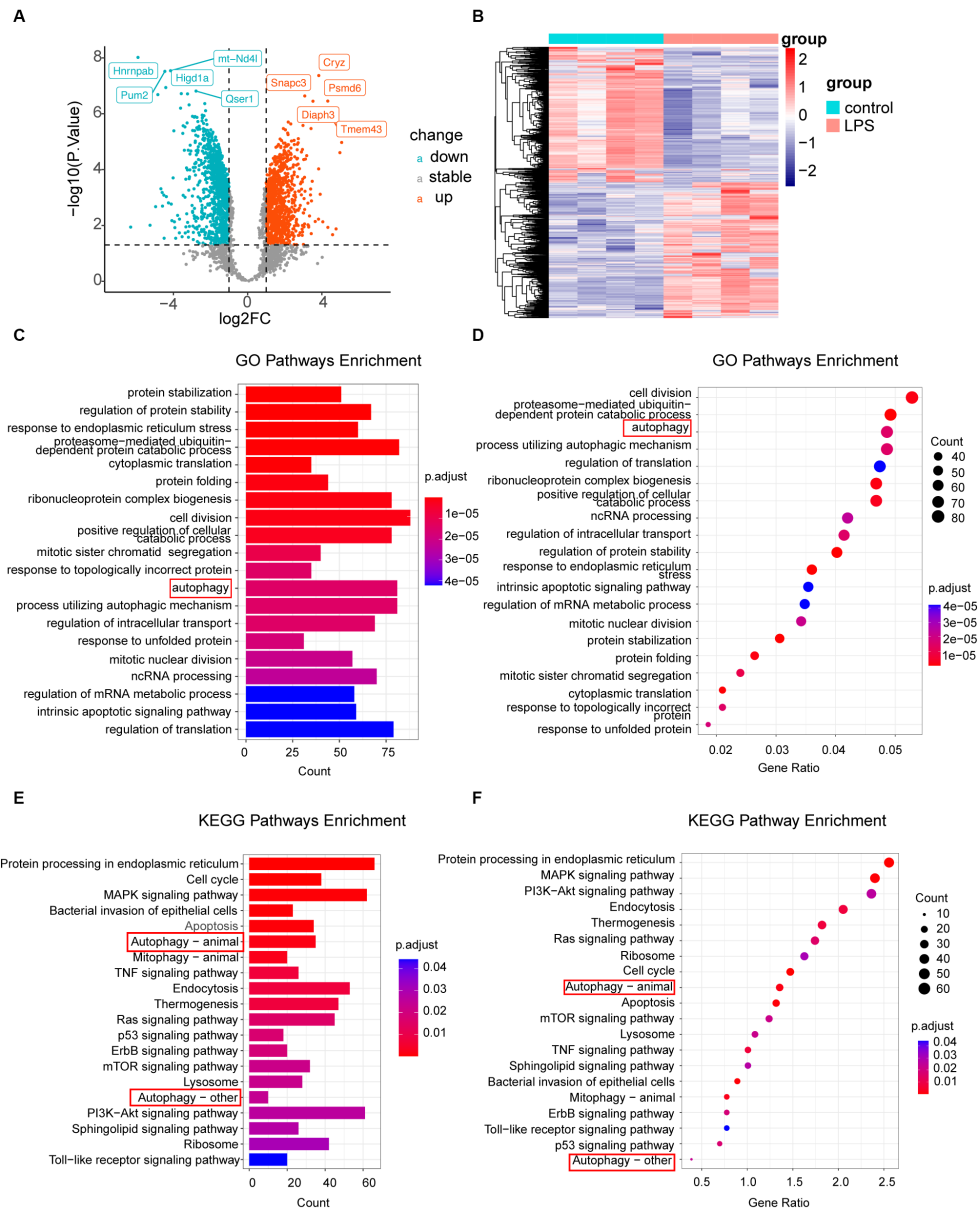
Analysis of DEGs. **(A)** Volcano plot illustrating differential gene expression screening. **(B)** Heatmap displaying genes with differential expression in LPS-treated samples compared to that in the control samples. **(C,D)** GO enrichment analysis of all genes, represented by column and bubble charts. **(E,F)** KEGG pathway enrichment analysis of different genes, shown in column and bubble charts, respectively. DEGs, differentially expressed genes; LPS, lipopolysaccharides; GO, Gene Ontology; KEGG, Kyoto Encyclopedia of Gene and Genome.

According to the criteria |log2FC| > 1 and *p*-value <0.05, 2,717 DEGs were identified in the dataset. Among these, 991 upregulated and 920 downregulated genes were observed in the LPS samples, as depicted by the volcano plot (Figure 2A). To gain a thorough understanding of the distinct expression patterns exhibited by DEGs among the various groups, we conducted an unsupervised hierarchical clustering analysis and generated heatmaps (Figure 2B). The heat map shows distinct clustering patterns among the samples. Furthermore, our GO and KEGG pathway enrichment analyses indicated that the autophagy pathway was significantly enriched in post-inflammatory astrocytes (Figures 2C–F). Additionally, we conducted separate GO enrichment analyses for the upregulated and downregulated genes and found that the autophagy pathway was prominently enriched among the upregulated genes (Supplementary material S1A–D). Moreover, we performed GSEA on the DEGs. GSEA, which ranked genes from the highest to the lowest fold change, revealed that the leading-edge subset, comprising genes involved in a specific pathway, was densely populated at the top of the ranking list. This indicates significant upregulation and suggests activation of the pathway. In this study, we observed that the autophagy pathway was upregulated after LPS stimulation, indicating its activation (Supplementary material S1E). These findings are consistent with those of previous studies (He et al., 2008; Duan et al., 2017; Fu et al., 2022; Zheng et al., 2023).

### 3.3 Autophagy-related DEGs differentiate LPS sample from the control sample

**Figure 3:**
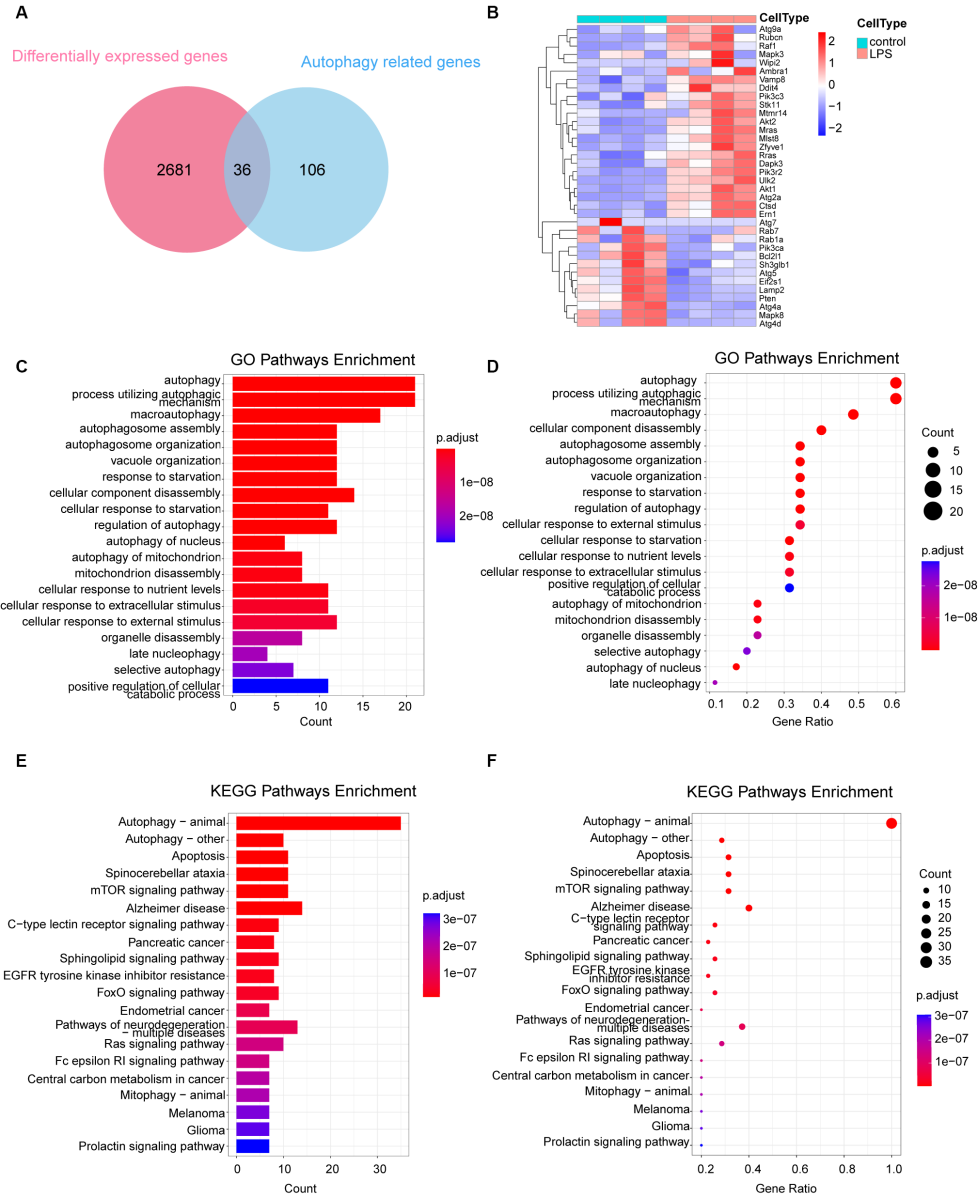
Analysis of 36 autophagy-related differentially expressed genes. **(A)** Venn diagram depicting the 36 autophagy-related DEGs. **(B)** Heatmap representing the expression of the 36 autophagy-related DEGs. **(C,D)** GO enrichment analysis of the 36 autophagy-related DEGs, illustrated in column and bubble charts. **(E,F)** KEGG pathway enrichment analysis of the 36 autophagy-related DEGs, depicted in column and bubble charts. DEGs, differentially expressed genes; LPS, lipopolysaccharides; GO, Gene Ontology; KEGG, Kyoto Encyclopedia of Gene and Genome.

A total of 142 autophagy-associated genes were acquired from the KEGG database following the elimination of redundant entries. To identify the intersection between the 2,717 differential genes and 142 autophagy genes, we utilized the Evenn platform, resulting in the identification of 36 intersecting genes (Figure 3A). These 36 genes were further analyzed and visualized using a heat map, which revealed 23 upregulated and 13 downregulated genes (Figure 3B). GO and KEGG enrichment analyses of these 36 autophagy-related genes confirmed their significant associations with autophagy, as indicated by the enriched pathways. These findings are presented using columns (Figures 3C,E) and bubbles (Figures 3D,F).

### 3.4 Analysis of hub genes

**Figure 4:**
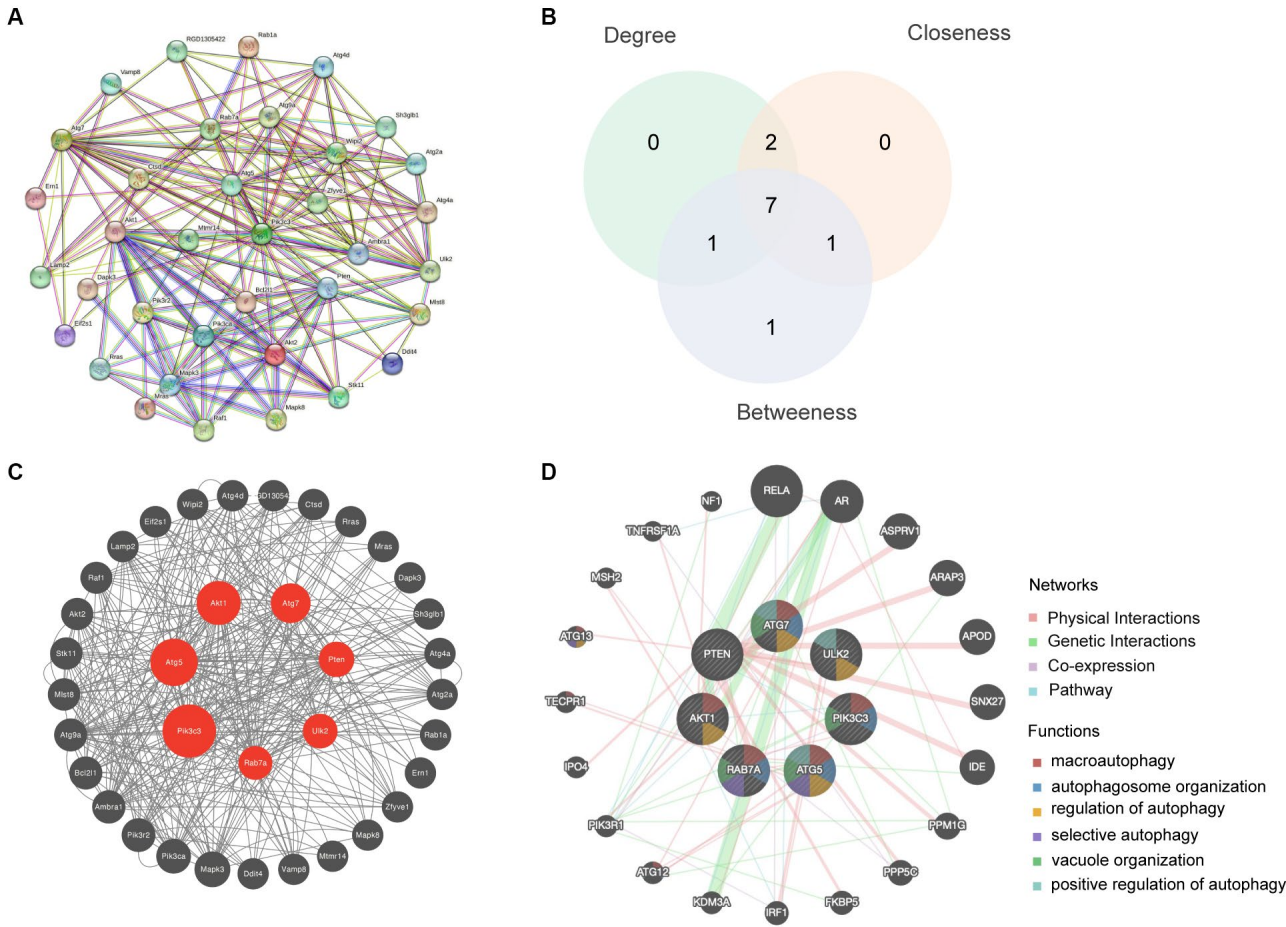
Hub gene analysis. **(A)** A PPI network based on the 36 autophagy-related genes was generated using the STRING database. **(B)** The top seven hub genes were identified by calculating the Betweenness, Closeness and Degree. **(C)** Hub genes were analyzed using the plugin cytoNCA. **(D)** Enrichment analysis of the seven hub genes was conducted using the GeneMANIA database.

The STRING database was used to create a PPI network by combining 36 autophagy-associated genes (Figure 4A). The top seven hub genes in the network were identified by assessing their Betweenness, Closeness, and Degree (Figure 4B). Subsequently, we analyzed hub genes using the CytoNCA plugin (Figure 4C). Enrichment analysis revealed that the top seven hub genes were significantly associated with microautophagy, autophagosome organization, and regulation of autophagy (Figure 4D).

### 3.5 Comparison of hub gene expression levels between the LPS-stimulated and control groups

**Figure 5:**
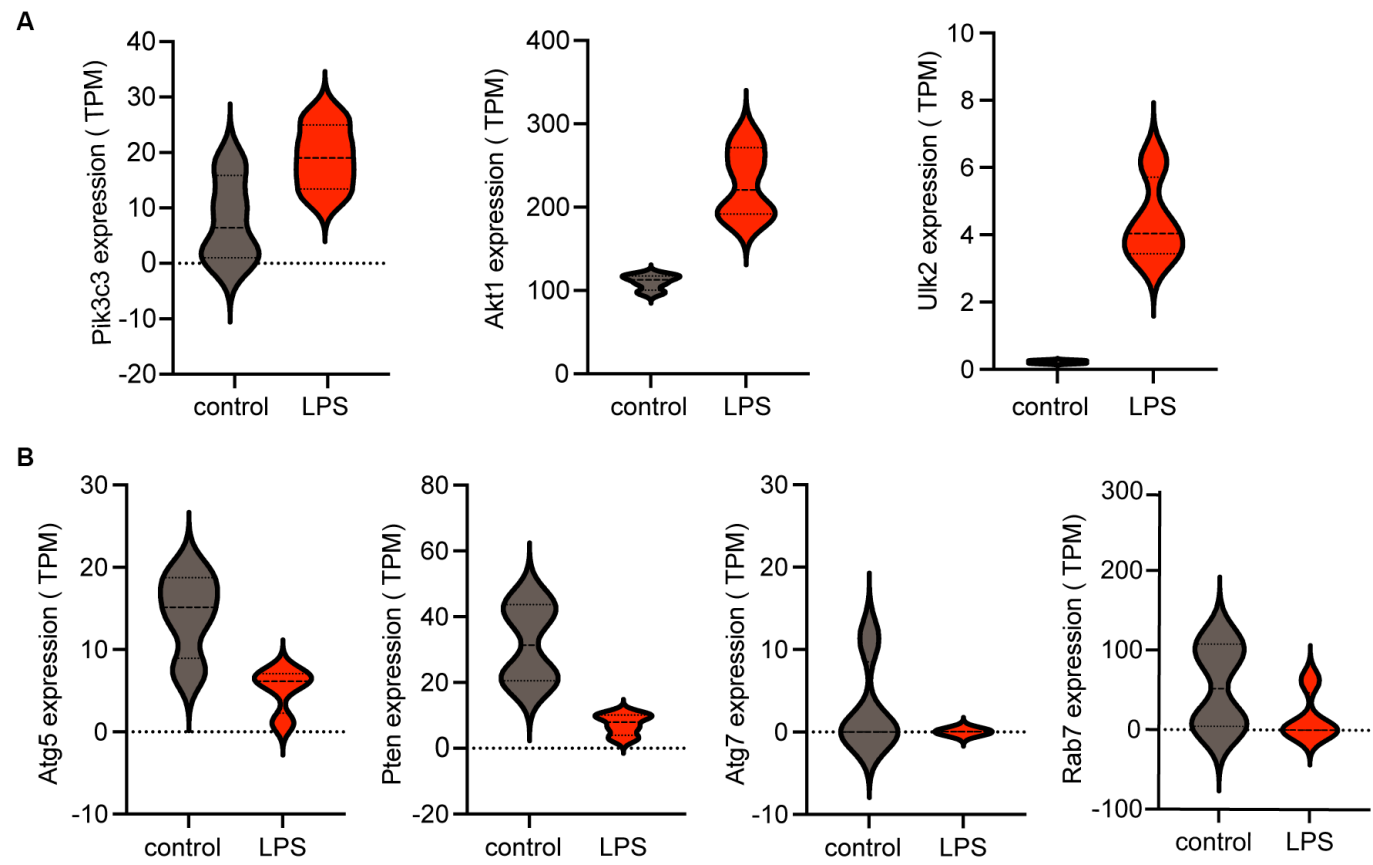
Expression of hub genes is shown in a violin plot. **(A)** Three upregulated autophagy-related genes (*Pik3c3, Akt1,* and *Ulk2*) among the seven hub genes. **(B)** Four downregulated autophagy-related genes (*Atg5, Pten, Atg7,* and *Rab7*) among the seven hub genes. TPM, transcripts per kilobase of exon model per million mapped reads; LPS, lipopolysaccharides.

The violin plot revealed altered expression levels of the seven hub genes in the LPS-stimulated group compared with those in the control group. Specifically, following LPS stimulation, *Pik3c3*, *Akt1*, and *Ulk2* were upregulated (Figure 5A), whereas *Atg5*, *Atg7*, *Rab7*, and *Pten* were downregulated (Figure 5B). However, it remains unclear whether these factors are upregulated or downregulated in ICH.

### 3.6 Expression of astrocyte markers in the ICH model and analysis of the association between astrocyte markers and genes relevant to autophagy

**Figure 6:**
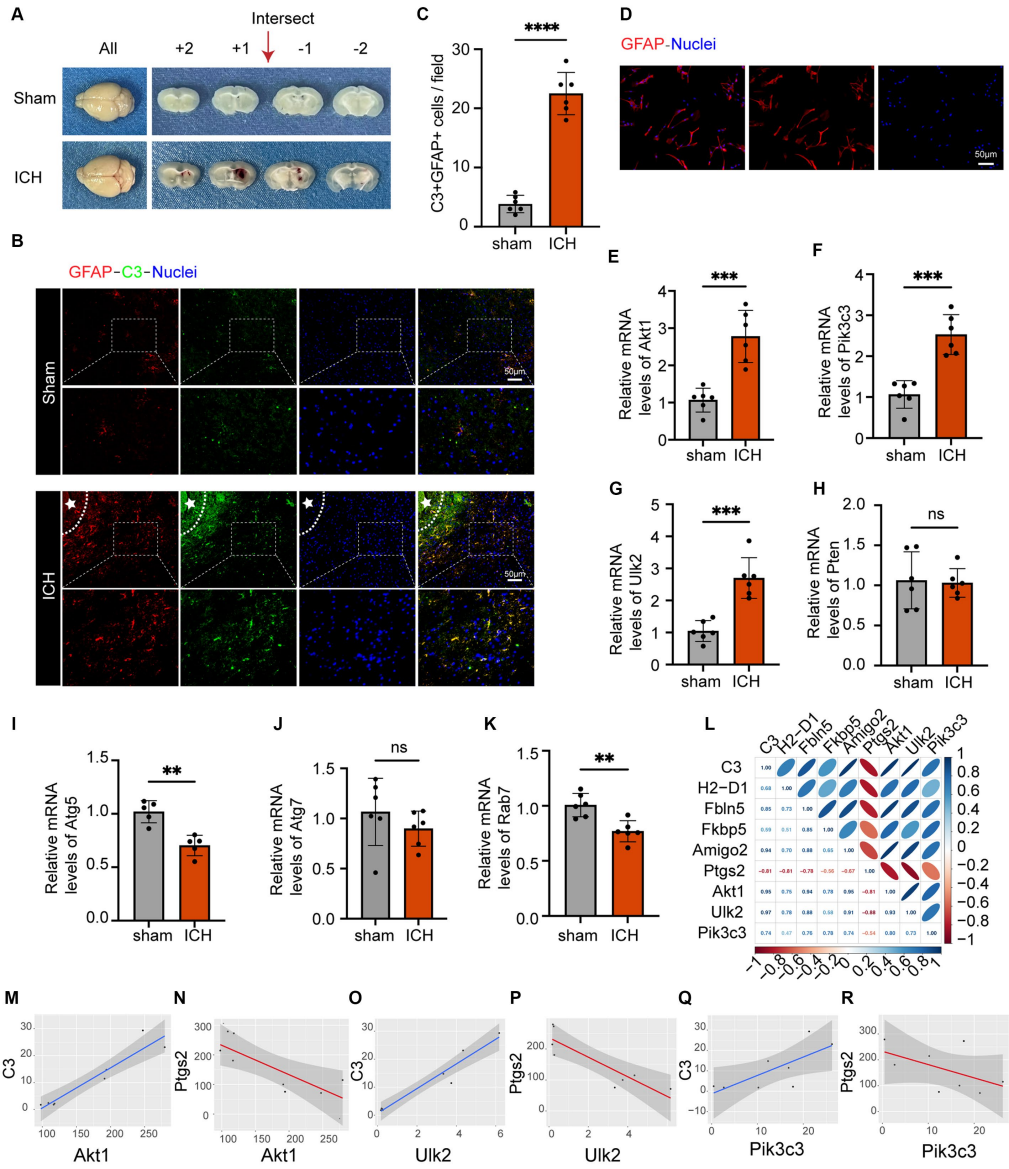
The astrocyte markers’ expression in the ICH model and the analysis of the association between astrocyte markers and genes relevant to autophagy. **(A)** Coronal brain sections of mice in the sham and ICH groups. **(B)** Double immunofluorescence staining for C3 and GFAP around the hematoma in the mice brain 24 h post-ICH. The white stars represent the location of the hematoma. The box outlined with white dotted lines represents the enlarged portion. Scale bar = 50 μm. **(C)** Quantification of C3 and GFAP expression and cell number per magnification. Data are presented as the mean ± SD; ^****^p < 0.0001 vs. sham group; *n* = 6. **(D)** GFAP immunofluorescence staining of isolated astrocytes. Scale bar = 50 μm. **(E–K)** qRT-PCR results showed that the expression levels of Akt1 (*p*-value = 0.0003), Pik3c3 (*p*-value = 0.0001), Ulk2 (*p*-value = 0.0002) were obviously higher and the expression levels of Atg5 (*p*-value = 0.001), and Rab7 (*p*-value = 0.0023) were lower, and the expression levels of Pten (*p*-value = 0.8459) and Atg7 (*p*-value = 0.3033) were not significantly altered after ICH. The black dots represent individual data in each group. All data are displayed as means ± SD; mean values for the control group were normalized to 1.0; ^**^p < 0.01, and ^***^p < 0.001 vs. sham group; *n* = 6. **(L)** Correlation analysis heat map. **(M–R)** Correlation lines of Akt1, Pik3c3 and Ulk2 with astrocytes of A1 and A2, respectively.

The ICH model is shown in Figure 6A. *In vivo* immunofluorescence analysis revealed significant upregulation of GFAP and C3 expression following ICH, accompanied by a noticeable increase in the ratio of C3 to GFAP. This indicated the robust activation of astrocytes with an inflammatory phenotype (Figures 6B,C). To verify the expression levels of these seven autophagy-related genes in astrocytes following ICH, we isolated astrocytes from the perihematomal area and performed qRT-PCR. Figure 6D shows fluorescence staining of isolated astrocytes, indicating that GFAP and the nucleus can merge completely. The same experiment was performed on the astrocyte cell line used for sequencing, which proved that the cells were not pollution-free (Supplementary material S1F). The PCR results showed that, compared to the control group, the expression of Pik3c3, Akt1, and Ulk2 in astrocytes significantly increased 24 h after ICH, and the expression of Atg5 and Rab7 in astrocytes decreased after ICH, while there was no significant difference between Pten and At7 (Figures 6L–R). As an increasing number of studies have demonstrated early autophagy activation after ICH, we considered the three upregulated factors as potential factors for improving ICH prognosis. In addition, we associated these three genes with the markers of A1 and A2 astrocytes (Figures 6G–M). Among these, C3, H2-D1, Fbln5, and FKBP5 are markers of A1 astrocytes, whereas Ptgs2 is a marker of A2 astrocytes (Liddelow et al., 2017). Notably, there was minimal expression in A2 astrocytes following LPS stimulation, with Ptgs2 as the sole marker present in the dataset. Furthermore, we observed positive correlations among Akt1, Ulk2, and Pik3c3 and A1 astrocytes, whereas they were negatively correlated with A2 astrocytes. Thus, we believe that these three factors can serve as targeted molecules for astrocytic autophagy following ICH, and provide valuable guidance for future research and treatment. Therefore, further investigation of their roles in ICH is of great significance for the diagnosis and management of this disorder.

### 3.7 Prediction of transcription factors, upstream miRNAs, and associated drugs linked to genes associated with autophagy

The Enrichr platform was used to predict the primary transcription factors, upstream miRNAs, and associated drugs linked to the three genes involved in autophagy. *FOXA2*, *CREB1*, and *SETDB1* were identified as key transcription factors based on data from the transcription factor PPIs database (Table 1). According to the miRTarBase database, Mmu-miR-700, mmu-miR-5122, and mmu-miR-1901 are the major upstream miRNAs (Table 2). In addition, the DSigDB database predicted several primary drugs such as 3-methyladenine CTD 00001217 and leupeptin CTD 00001574 (Table 3). The identification of these transcription factors, miRNAs, and drugs has significant implications for ICH progression. Furthermore, these predicted drugs have potential therapeutic applications for the treatment of ICH.

**Table 1:** Transcriptional factor targets of three autophagy-related genes in ICH.

Term	*p*-value	Adjusted *p*-value	Odds ratio	Combined score
FOXA2	0.00224839	0.02323331	713.678571	4351.68576
CREB1	1.33 × 10^−4^	0.00212484	300.984848	2686.16101
SETDB1	1.37 × 10^−4^	0.00212484	296.462687	2637.00541
GATA2	0.00404468	0.03134625	384.057692	2116.29358
IRF1	0.00673508	0.03154705	226.738636	1133.78972
PPARGC1A	0.00956969	0.03154705	158.206349	735.52576
TAL1	0.00971873	0.03154705	155.726563	721.590219
GATA1	0.01031474	0.03154705	146.536765	670.285691
SOX2	0.01046371	0.03154705	144.405797	658.467663
IRF3	0.01120831	0.03154705	134.614865	604.568747

**Table 2:** MicroRNA targets of three autophagy-related genes in ICH.

Term	*p*-value	Adjusted *p*-value	Odds ratio	Combined score
mmu-miR-700	0.01626173	0.18068138	92.0787037	379.266738
mmu-miR-5122	0.01670679	0.18068138	89.5765766	366.541985
mmu-miR-1901	0.01981846	0.18068138	75.2462121	295.051037
hsa-miR-4738-5p	0.00572043	0.18068138	43.1909605	223.025639
hsa-miR-564	0.01015842	0.18068138	31.7217538	145.585488
hsa-miR-3610	0.04243706	0.27101173	34.4597902	108.883743
mmu-miR-380-3p	0.01489715	0.18068138	25.7158697	108.176
hsa-miR-4473	0.01633582	0.18068138	24.4335757	100.529387
hsa-miR-3613-5p	0.01641992	0.18068138	24.3638761	100.11751

**Table 3:** Drug targets of three autophagy-related genes in ICH.

Term	*p*-value	Adjusted *p*-value	Odds ratio	Combined score
Tetradioxin CTD 00006848	0.006682774	0.019042345	48,696	243880.3814
3-methyladenine CTD 00001217	5.26 × 10^−6^	0.002067378	1597.76	19421.22606
(5-methyl-3-(morpholinomethyl)-2,3-dihydro-[1,4]oxazino[2,3,4-hi]indol-6-yl)(naphthalen-1-yl)methanone CTD 00002747	0.001649143	0.017734459	999.35	6403.334808
Leupeptin CTD 00001574	0.001649143	0.017734459	999.35	6403.334808
butein CTD 00001872	0.001649143	0.017734459	999.35	6403.334808
Tipifarnib CTD 00003753	0.001649143	0.017734459	999.35	6403.334808
WP1066 CTD 00004704	0.001649143	0.017734459	999.35	6403.334808
Perifosine CTD 00003447	0.001649143	0.017734459	999.35	6403.334808
Triciribine CTD 00001109	0.001649143	0.017734459	999.35	6403.334808
Eckol CTD 00002503	0.001649143	0.017734459	999.35	6403.334808

## 4 Discussion

Emerging data indicates that inflammation may have a crucial effect on the progression of secondary brain damage following ICH (Wang and Doré, 2007; Zhou et al., 2014; Zhu et al., 2019). In response to ICH, inflammatory reactions occur rapidly, leading to the activation of neuroglia and infiltration of leukocytes, which release proinflammatory substances (Fu et al., 2015; Urday et al., 2015; Murthy et al., 2016). Astrocytes play a significant role in central neuroinflammation by secreting proinflammatory cytokines, chemokines, free radicals, and toxic compounds. This release may lead to cerebral edema, disturbance of the blood-brain barrier, and neuronal apoptosis. However, astrocyte activation hinders the entry of immune cells from outside the CNS and enhances the stability of the blood-brain barrier by generating sonic hedgehogs (Shh) (Alvarez et al., 2011). Additionally, studies have shown that astrocytes may undergo harmful changes when exposed to bacterial lipopolysaccharides during inflammation (Jiang et al., 2021). This transformation leads to the release of neurotoxic substances such as complement components and inflammatory cytokines, which are responsible for neuronal and oligodendrocyte cell death.

In this study, we developed an inflammatory model by stimulating astrocytes with lipopolysaccharide (LPS). Subsequent enrichment analysis of the sequencing data revealed a significant activation of the autophagic pathway. Differential gene analysis was performed, and autophagy-related genes were intersected, leading to the identification of Pik3c3, Akt1, and Ulk2 as target genes for astrocyte autophagy in response to inflammation after ICH.

Autophagy is a vital mechanism in eukaryotic cells that is responsible for the breakdown of cytoplasmic proteins and organelles damaged by lysosomes. This process is regulated by autophagy-related genes. It plays a crucial role in numerous physiological and pathological processes, including cellular balance, aging, immune responses, tumor development, and neurodegenerative disorders. Previous studies have proposed that autophagy is promptly initiated following ICH (He et al., 2008). For instance, Duan et al. (2017) revealed that the overactivation of autophagy could potentially contribute to brain damage caused by endoplasmic reticulum stress within 6 h of ICH. Additionally, Yuan et al. found that microglial autophagy is activated 3 h after ICH and contributes to erythrocyte lysis-induced microglial inflammatory damage in neuronal cells (Yuan et al., 2017). Consistent with these findings, our PCR analysis showed a significant upregulation of three autophagy-related genes, *Pik3c3*, *Akt1*, and *Ulk2*, in astrocytes following ICH compared to the sham group. However, research on the role of astrocytic autophagy in post-ICH inflammation is limited. This study aimed to bridge this gap and provide valuable insights for future investigations of the relationship between astrocyte autophagy and post-hemorrhagic inflammatory responses.

Enrichment analysis of the data demonstrated a noteworthy association between autophagy and DEGs. By intersecting DEGs with autophagy-related genes, we identified 36 autophagy-associated genes. Subsequently, we calculated the Betweenness, Closeness, and Degree using the Cytoscape software and identified seven hub genes—*Pik3c3*, *Atg5*, *Atg7*, *Akt1*, *Rab7*, *Pten*, and *Ulk2*. Analysis of PPI identified seven central genes from a pool of 36 autophagy-related genes. In addition, through an extensive literature review, we established that autophagy is upregulated after ICH. In addition to experimental verification by astrocyte PCR after ICH, we selected three astrocyte markers that significantly upregulated autophagy in the inflammatory state after ICH: Pik3c3, Akt1, and Ulk2. The *Pik3c3* gene is responsible for synthesizing the catalytic subunit type 3 of phosphatidylinositol 3-kinase, which plays a crucial role in initiating autophagy (Volinia et al., 1995). Previous studies have suggested that Pik3c3 is widely expressed in the CNS and its removal results in synaptic loss, extensive gliosis, and progressive neurodegeneration (Wang et al., 2011). Yang et al. (2021a,b, 2022) discovered that autophagy is impaired in T, dendritic, and myeloid cells lacking Pik3c3. Mice with a specific deficiency of Pik3c3 in dendritic cells showed significant resistance to experimental autoimmune encephalomyelitis, which was linked to the reduced reactivation of initiating T cells within the CNS. However, Pik3c3 expression has not yet been reported in patients with ICH. The RAC-alpha serine/threonine-protein kinase encoded by Akt1 is involved in numerous cellular functions, including growth, proliferation, survival, and angiogenesis. Alterations in Akt1 expression gene have been detected in various cancers (George et al., 2022). Akt1 has been investigated as a potential therapeutic target in ICH. Some studies have proposed that Akt1 exerts a protective effect against ICH by facilitating neuronal viability and mitigating inflammatory responses (Lee et al., 2009). However, the specific function of Akt1 in ICH remains unclear, and additional research is required to comprehensively understand its mechanisms and potential therapeutic uses. Ulk2 is a crucial component of the Ulk complex and is responsible for the initiation of autophagy (Shi X. et al., 2020). Ulk2 functions upstream of the Pik3c3 to regulate the development of autophagophores, which are precursors of autophagosomes (Mizushima, 2010; Harris et al., 2022). While the role of Ulk2 in ICH has not been extensively studied, its potential significance in ICH pathology can be inferred from its involvement in autophagy and maintenance of neuronal balance. Therefore, the 36 autophagy-related genes identified in this study may play crucial roles in ICH via these pathways.

The present study also identified three autophagy-related DEGs corresponding to transcription factors, upstream miRNAs, and drugs. The primary transcription factors identified were FOXA2, CREB1, and SETDB1. FOXA2 is an important regulator of nerve production during embryonic development. Studies have shown that in a rat model of Parkinson’s disease, co-transplantation of neural stem/progenitor cells (NSCs/NPCs) with midbrain-derived astrocytes overexpressing the transcription factors NURR1 and FOXA2 promotes graft maturation and survival, thereby improving treatment outcomes (Tsai, 2018). However, Foxa2 expression has not been reported in patients with stroke. Cyclic-AMP response binding protein (CREB), composed of 341 amino acid residues, is a member of the leucine-rich zipper structure (bZIP) superfamily. CREB is a key transcription factor that regulates CNS functions and is involved in neuroprotection and neurodevelopment (Wu et al., 2019; Liu et al., 2021; Yan et al., 2022). Studies have shown that members of the CREB family are essential for the survival of neurons in the body. The absence of CREBs and CREMs in the developing brain leads to widespread cell death, and postnatal transcriptional disruptions mediated by CREBs or CREMs trigger selective and progressive neurodegeneration (Mantamadiotis et al., 2002). Some studies have shown that two pathways, CCR5/PKA/CREB/NLRP1 signaling pathway and CREB/Nr4a1/NF-κB pathway, are related to inflammatory and neuroprotective effects after ICH (Wu et al., 2019; Yan et al., 2021). The CERB pathway plays a pivotal role in ICH; however, the precise mechanism remains unclear. SETDB1 is an enzyme responsible for modifying histone lysine, which plays a crucial role in regulating gene expression and maintaining the cellular balance necessary for the proper function of the nervous system. Additionally, its involvement has been observed in the development of various CNS disorders, such as brain tumors, schizophrenia, Huntington’s disease, and autism spectrum disorders (Markouli et al., 2021). SETDB1 has recently gained recognition as a protein that aids cancer cells in evading the immune system and holds potential as an novel target for cancer treatment (Griffin et al., 2021). However, it has not been reported in relation to ICH or stroke. The three miRNAs identified were mmu-miR-700, mmu-miR-5122, and mmu-miR-1901. None of these three factors was found to be associated with ICH, proving that this is a new field that needs to be explored. Among the predicted drugs, 3-methyladenine (3-MA) is a commonly used pharmacological inhibitor of autophagy. By inhibiting autophagy, 3-methyladenosine can be used to investigate the function and significance of autophagy in diverse biological processes and diseases. It is frequently employed to explore the involvement of autophagy in cellular responses such as cell survival, cell death, and regulation of disease-related pathways. It may be possible to improve associated inflammatory symptoms by reducing autophagy after ICH. Leupeptin, a protease inhibitor with wide-ranging activities and the ability to permeate cellular membranes, effectively inhibits serine, cysteine, and threonine proteases. According to Liew et al. (2019), ICH triggers rapid overactivation of proteasomes, leading to the aggravation of ER stress/proteostasis disruption. Thus, leupeptin is a promising therapeutic agent for treating inflammation after ICH.

While this study primarily focused on the role of astrocytic autophagy in the aftermath of ICH, we must not overlook the contributions of other glial cells and the interplay between astrocytes and these cells. For instance, microglia, the primary phagocytic cells of the brain tissue, also play a crucial role in the process of brain injury following hemorrhage. The polarization state of microglia can influence hematoma absorption and the formation of perihematomal edema, which correlates with the prognosis of ICH (Lan et al., 2017; Zhang et al., 2017). Bidirectional communication between astrocytes and microglia, mediated by the secretion of various cytokines and inflammatory mediators, regulates inflammation in the CNS. According to research findings, IL-1α, TNF-α, and C1q are released by activated microglia. This leads to the release of the neurotoxic complement C3d by A1 reactive astrocytes, resulting in the rapid demise of neurons and mature differentiated oligodendrocytes (Liddelow et al., 2017). On the other hand, astrocytes release cytokines such as IL-1β and IL-6, which can have both proinflammatory and anti-inflammatory effects. This allows them to regulate microglial activation and function (Scimemi, 2018). Similarly, reciprocal communication between astrocytes and oligodendrocytes is vital for the immune regulation of the CNS. Oligodendrocytes possess diverse receptors to detect inflammatory signals released by astrocytes, and vice versa (Omari et al., 2005; Ramesh et al., 2012; Moyon et al., 2015). Although evidence indicates a close functional relationship between astrocytes and oligodendrocytes, further exploration is required to fully understand the exact mechanisms of their interaction. To make significant contributions to disease therapeutics, it is crucial for future studies to conduct a comprehensive and specific analysis of how astrocytes interact with other cells in the CNS, thus gaining a deeper understanding of the neuroglial cell network.

## 5 Conclusion

In this study, the analysis of differentially expressed genes in conjunction with autophagy-related genes led to the identification of 36 DEGs. Further examination facilitated the identification of three crucial autophagy-related genes. These findings contribute to a comprehensive understanding of astrocyte autophagy in ICH and provide novel perspectives for potential therapeutic approaches to address this condition. Additionally, 3-methyladenine and leupeptin are promising drug candidates for ICH treatment.

## Data availability statement

The datasets presented in this study can be found in online repositories. The names of the repository/repositories and accession number(s) can be found in the article/Supplementary material.

## Ethics statement

The animal study was approved by Medical Ethics Committee at the First Hospital of Soochow University. The study was conducted in accordance with the local legislation and institutional requirements.

## Author contributions

YZ: Writing – review & editing, Data curation, Methodology, Project administration, Writing – original draft. CD: Methodology, Writing – original draft, Conceptualization, Funding acquisition. HY: Investigation, Project administration, Writing – review & editing. GJ: Writing – review & editing, Data curation, Formal analysis, Methodology, Software. HS: Software, Resources, Validation, Writing – original draft. HL: Resources, Software, Data curation, Project administration, Writing – review & editing. ZW: Project administration, Conceptualization, Methodology, Writing – original draft. XZ: Methodology, Data curation, Supervision, Writing – review & editing. XL: Data curation, Writing – review & editing, Conceptualization, Funding acquisition, Project administration, Writing – original draft. MH: Conceptualization, Funding acquisition, Writing – review & editing, Validation, Writing – original draft.

## Footnotes

^1^ https://www.ensembl.org/index.html

^2^ https://www.kegg.jp

^3^ http://www.ehbio.com/
