# Supplementary material for: Transcriptomic analysis reveals novel hub genes associated with astrocyte autophagy in intracerebral hemorrhage
Source: Front Aging Neurosci. 2024 Jul 3;16:1433094. doi: 10.3389/fnagi.2024.1433094 (PMC11256209; doi:10.3389/fnagi.2024.1433094)
Supplement: Supplementary file 1 [file Table_1.DOCX]

data4<-GSE合并lamp2_a1_a2_mbp相关性

data3<-t(data4)

data3 <- as.data.frame(data3)

Lamp2<-data4[,2]

Mbp<-data4[,4]

Mag<-data4[,3]

Mog<-data4[,5]

Plp1<-data4[,6]

C3<- data4[,7]

S100a10<- data4[,21]

H2.D1<- data4[,2]

Fbln5<- data4[,3]

Fkbp5<-data4[,4]

Amigo2<-data4[,5]

Ptgs2<-data4[,6]

Ulk2<-data4[,8]

Pik3c3<-data4[,9]

Lamp2<-data.frame(Lamp2)

Mbp<-data.frame(Mbp)

H2.D1<-data.frame(H2.D1)

Fbln5<- data.frame(Fbln5)

Fkbp5<-data.frame(Fkbp5)

Amigo2<-data.frame(Amigo2)

Ptgs2<-data.frame(Ptgs2)

Ulk2<-data.frame(Ulk2)

Pik3c3<-data.frame(Pik3c3)

data3<-read.csv(file = '三因子和A1 A2 星胶.csv')

C3<-data.frame(C3)

Akt1 <- data4[,7]

Akt1<-data.frame(Akt1)

cor.test (Lamp2, Mag, method="spearman")

cor.test (C3, Pik3c3, method="spearman")

cor.test (H2.D1,Pik3c3, method="spearman")

cor.test (Fbln5, Pik3c3,method="spearman")

cor.test ( Fkbp5,Pik3c3, method="spearman")

cor.test ( Amigo2,Pik3c3, method="spearman")

cor.test ( Ptgs2, Pik3c3,method="spearman")

library(ggplot2)

p1 <- ggplot(data3, aes(x = Lamp2, y =Mbp))

p2 <- p1 + geom_point()

p3 <- p2 + geom_smooth(method="lm")

p3

library(ggpubr)

ggscatter(data4, x = "Lamp2", y = "Mbp",

add = "reg.line",conf.int = TRUE,

fill = "lightgray")

#加上P值

ggscatter(data4, x = "Lamp2", y = "Mbp",

color = "red3",fill = "lightgray",

add = "reg.line", conf.int = TRUE,

add.params = list( color = "black",fill = "lightgray",fill = "lightgray"),

cor.coef = T,

cor.coeff.args = list(),

cor.method = "pearson",

cor.coef.coord = c(3500, 150000),

cor.coef.size = 8)

data3$group <- rep(c("sham", "ICH"), each = 3)

# 使用不同的颜色绘制不同组别的点

ggscatter(data4, x = "Lamp2", y = "Mag",

color = "group", palette = c("pink", "Sky Blue"),

fill = "lightgray",

add = "reg.line", conf.int = TRUE,

add.params = list(color = "black", fill = "lightgray"),

cor.coef = TRUE,

cor.coeff.args = list(),

cor.method = "pearson",

cor.coef.coord = c(3500, 150000),

cor.coef.size = 8)

ggscatter(data4, x = "Lamp2", y = "Mbp",

color = "group", palette = c("pink", "Sky Blue"),

shape = "source", legend = "right",

fill = "lightgray",

add = "reg.line", conf.int = TRUE,

add.params = list(color = "black", fill = "lightgray"),

cor.coef = TRUE,

cor.coeff.args = list(),

cor.method = "pearson",

cor.coef.coord = c(3500, 150000),

cor.coef.size = 8)

library(corrplot)

library(ggplot2)

library(ggpubr)

data3<-data3[,-1]

class(data3)

library(corrplot)

cor (data3, method="pearson")

data3c <- cor (data3, method="pearson")

corrplot(data3c)

data3<-t(data3)

corrplot(data3c, method = "circle",

tl.col = "black", tl.cex = 1.2, tl.srt = 45)

corrplot(data3c, method = "ellipse",

type = "upper",

tl.col = "black", tl.cex = 1.2, tl.srt = 45

)

corrplot(data3c, method = "ellipse", type = "upper",

tl.col = "black", tl.cex = 0.8, tl.srt = 45,tl.pos = "lt")

corrplot(data3c, method = "number", type = "lower",

tl.col = "n", tl.cex = 0.8, tl.pos = "n",

add = T)

addcol <- colorRampPalette(c("red", "white", "blue"))

corrplot(data3c, method = "pie", type = "upper",col = addcol(100),

tl.col = "black", tl.cex = 0.8, tl.srt = 45,

tl.pos = "lt")

corrplot(data3c, method = "number", type = "lower",col = addcol(100),

tl.col = "n", tl.cex = 0.8, tl.pos = "n",

add = T)

testRes = cor.mtest(data3, method="pearson",conf.level = 0.95)

corrplot(data3c, method = "color", col = addcol(100),

tl.col = "black", tl.cex = 0.8, tl.srt = 45,tl.pos = "lt",

p.mat = testRes$p, diag = T, type = 'upper',

sig.level = c(0.001, 0.01, 0.05), pch.cex = 1.2,

insig = 'label_sig', pch.col = 'grey20', order = 'AOE')

corrplot(data3c, method = "number", type = "lower",col = addcol(100),

tl.col = "n", tl.cex = 0.8, tl.pos = "n",order = 'AOE',

add = T)

install.packages("PerformanceAnalytics")

library(PerformanceAnalytics)

library(tidyverse)

library(future.apply)

library(survival)

setwd("/home/data/zhengyun/autophagy myself")

getwd()

df <- read.csv("自噬&amp;组学 36交集基因data.csv")

data<-read.csv("自己组学数据.csv")

exprSet=data3

singleGene_cor <- function(gene){

y <- as.numeric(exprSet[gene,])

rownames <- rownames(exprSet)

do.call(rbind,future_lapply(rownames, function(x){

dd <- cor.test(as.numeric(exprSet[x,]), y, type='spearman')

data.frame(gene=gene,mRNAs=x,cor=dd$estimate,p.value=dd$p.value)

}))

}

dd <- singleGene_cor('Pik3c3') #如果更换基因，只用更新基因名即可。

write.table(dd, file = "Pik3c3 cor.txt",sep = "\t",row.names = T,col.names = NA,quote = F)

write.csv(dd, file = "Pik3c3 cor.csv")

library(dplyr)

library(tidyr)

corSig <- dd %>%

filter(p.value < 0.05) %>%

arrange(desc(abs(cor)))%>%

dplyr::slice(1:420)
