# Supplementary figures and images for: Transcriptomic analysis reveals novel hub genes associated with astrocyte autophagy in intracerebral hemorrhage
Source: Front Aging Neurosci. 2024 Jul 3;16:1433094. doi: 10.3389/fnagi.2024.1433094 (PMC11256209; doi:10.3389/fnagi.2024.1433094)

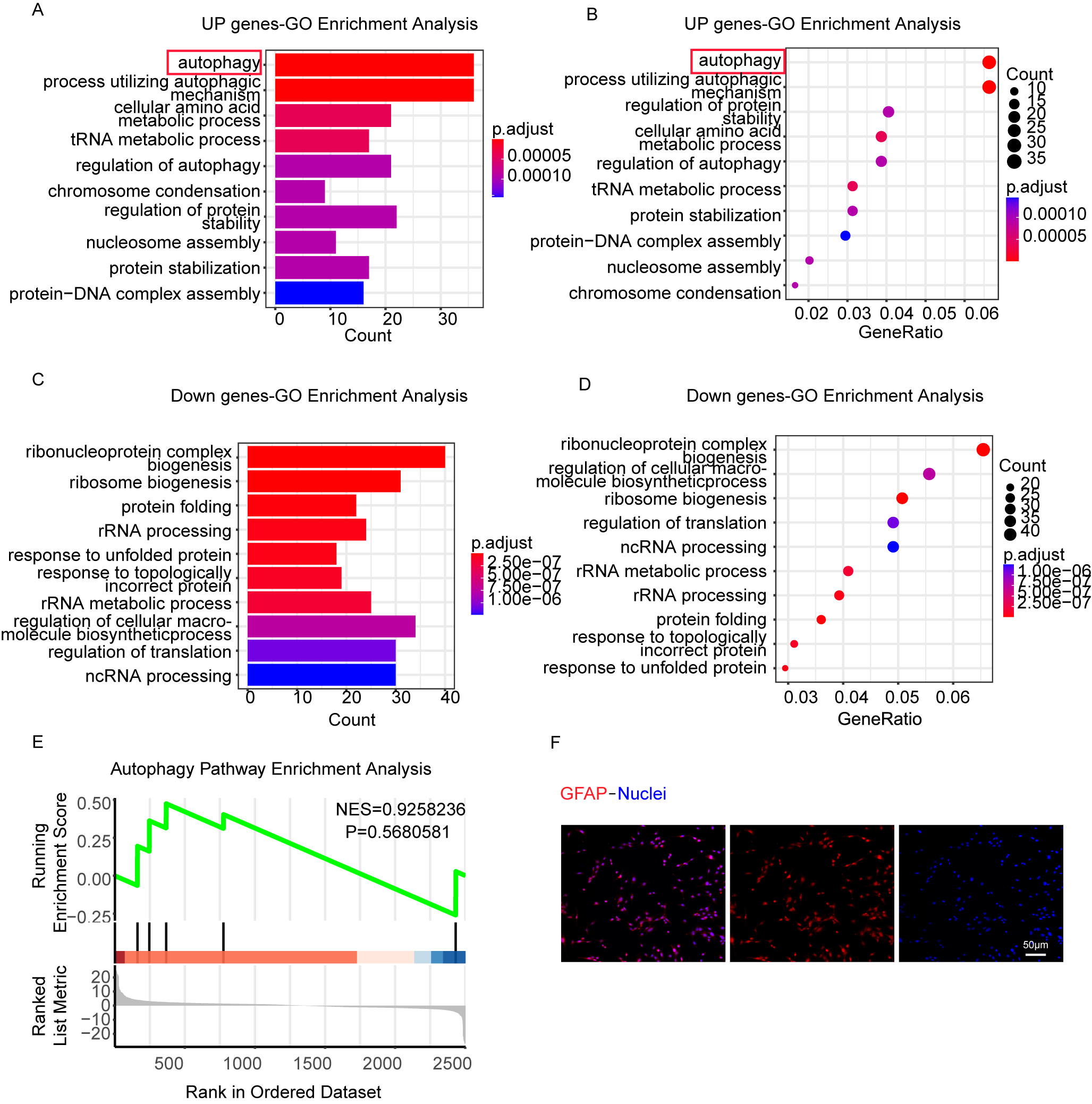

Supplement: Supplementary file 5 [file Image_1.TIF]

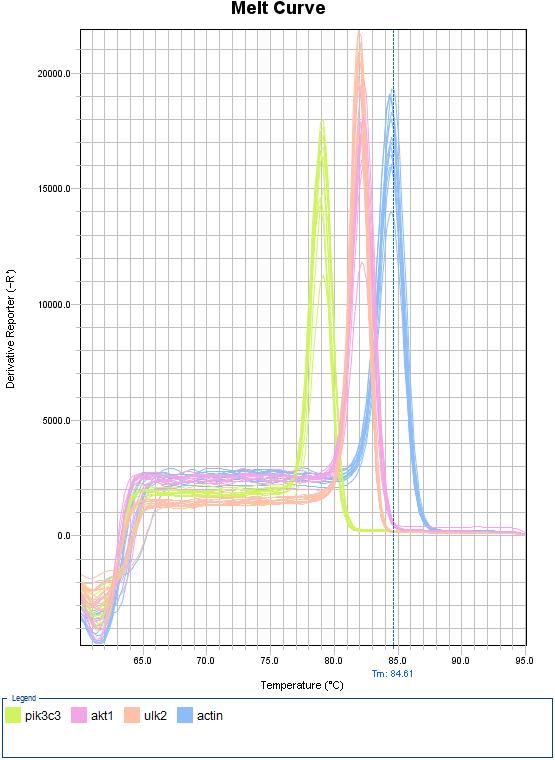

Supplement: Supplementary file 6 [file Image_2.JPEG]

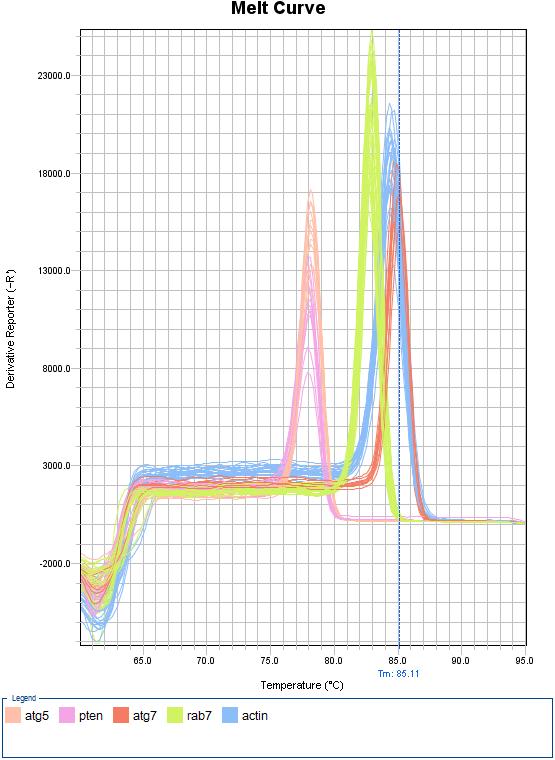

Supplement: Supplementary file 7 [file Image_3.JPEG]

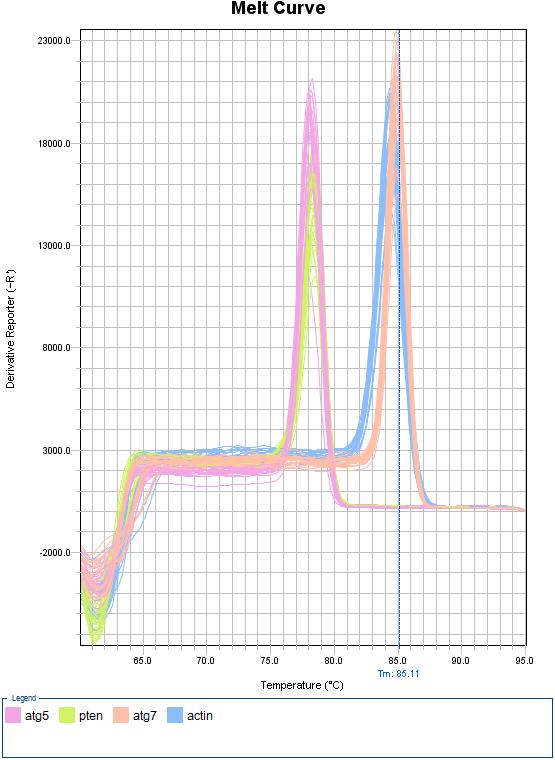

Supplement: Supplementary file 8 [file Image_4.JPEG]
